# AI-Assisted Molecular Structure Prediction from Tandem Mass Spectra: Reliability and Validation

**DOI:** 10.3390/ijms272110012

**Published:** 2026-11-08

**Authors:** Jun Sang Yu, Sung Hue Jeong, Yejin Kim, Dong Keun Lee, Hye Hyun Yoo

**Affiliations:** 1 College of Pharmacy, Jeju National University, Jeju 63243, Republic of Korea; yujs0804@jejunu.ac.kr; 2 Pharmacomicrobiomics Research Center, College of Pharmacy, Hanyang University, Ansan 15588, Republic of Korea; 3 GENECT, Konkuk University Campus Town, Seoul 05073, Republic of Korea

**Keywords:** artificial intelligence, tandem mass spectrometry, structure prediction, database retrieval, molecular generation

## Abstract

Artificial intelligence (AI) has broadened the interpretation of tandem mass spectra (MS/MS) beyond experimental library matching, enabling molecular fingerprint prediction, candidate ranking, and generation of previously unlisted structures. Yet these outputs do not all support the same claim about chemical identity. This review briefly summarizes database-restricted retrieval, database-free generation, and scaffold-guided hybrid methods, and then asks what additional evidence is needed to turn a plausible structure into a defensible annotation. We examine failure modes involving incomplete candidate spaces, erroneous precursor assignments, structurally related isomers, leakage between training and test data, and misinterpreted confidence scores. The review contributes two practical elements: a candidate-discrimination workflow, in which alternative structures are retained and targeted measurements such as multienergy fragmentation, retention behavior, ion mobility, and authentic standards are selected according to the unresolved structural question; and an evidence-to-claim checklist that links each question to the most informative measurement and to the strongest supportable identification confidence level. Both synthesize established analytical principles for small-molecule electrospray LC–HRMS/MS rather than constituting a validated scoring algorithm. By making the basis of each annotation transparent, they are intended to improve the practical value of AI-assisted MS/MS in metabolomics, pharmaceutical analysis, environmental screening, and forensic toxicology.

## 1. Introduction

High-resolution tandem mass spectrometry (HRMS/MS) has become a principal approach to investigating small molecules for which reference materials or reference spectra may be unavailable. Accurate precursor masses, isotope patterns, fragment-ion formulas, and neutral losses constrain candidate structures, but usually do not encode an unambiguous molecular graph. Ion formation and gas-phase rearrangements can obscure the relation between precursor structure and observed products; positional isomers and closely related analogues may yield nearly indistinguishable spectra. Accordingly, the analytical act of proposing a candidate must be separated from demonstrating that the candidate is the compound present in a sample [1,2,3].

Artificial intelligence (AI) and related computational methods now operate throughout this process. In silico fragmentation and chemical database searching rank known structures, learned molecular fingerprints extend retrieval beyond measured reference libraries, and generative systems propose molecules not explicitly contained in a candidate database [4,5,6,7,8,9,10,11,12,13,14,15,16,17]. The field has therefore progressed from finding a familiar spectral match toward producing plausible structural explanations for large regions of incompletely annotated chemical space. Such progress is especially relevant for unexpected drug metabolites, emerging synthetic analogues, and trace environmental transformation products.

**Figure 1:**
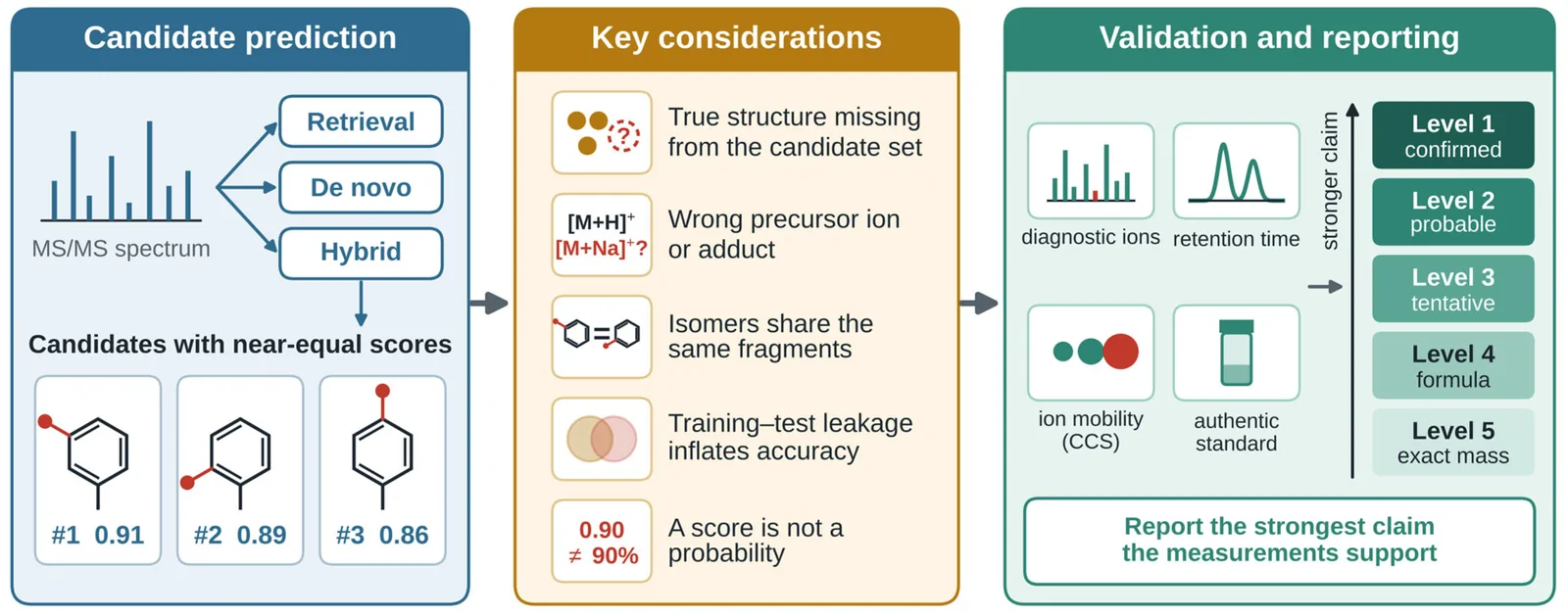
Overview of AI-assisted structure prediction from MS/MS and its analytical validation. Retrieval, de novo, and hybrid models propose ranked candidates that may differ only in substitution position (**left**). Key considerations explain why a top-ranked candidate can be wrong (**middle**). Discriminating measurements determine the strongest identification confidence level that can be reported [2] (**right**). CCS, collision cross section.

The literature already contains authoritative surveys of this field. Hupatz et al. critically reviewed in silico methods for structural annotation in LC–HRMS non-targeted screening [18], Schneider et al. reviewed de novo structure prediction algorithms, benchmarks, and limitations [19], and Hong et al. reviewed machine learning across small-molecule mass spectrometry [20]. Their emphasis is on how candidates are generated, ranked, and benchmarked; less attention is given to which identity claim a given combination of computational output and measurement can support, and to how the next experiment should be chosen when several candidates remain. This review addresses that interface and contributes two elements: a candidate-discrimination workflow (Section 4.1) and an evidence-to-claim checklist mapped onto the identification confidence levels of Schymanski et al. [2]. Our scope is small-molecule MS/MS, principally electrospray LC–HRMS/MS; gas chromatography with electron ionization, proteomics, and native mass spectrometry are not covered. We briefly organize computational advances by their underlying task, identify the most consequential sources of false certainty, and then describe the workflow (Figure 1). The workflow was derived from the cited literature and from the authors’ experience with non-targeted annotation of drug metabolites [21,22] and forensic samples; it is a synthesis of established analytical principles, not a newly validated algorithm or quantitative confidence score [2,23,24,25].

The evidence required for a structural claim also depends on its use. In metabolomics and environmental non-targeted analysis, the Metabolomics Standards Initiative (MSI) levels and the Schymanski scale communicate confidence, and tentative structures are routinely reported [1,2]. In forensic science, an identification used for legal purposes is expected to meet defined identification criteria: seized-drug analysis combines analytical techniques based on different chemical or physical properties [26], and forensic toxicology specifies minimum identification criteria that include comparison with a reference material analyzed under the same conditions [27]; an AI-ranked candidate can guide this work but cannot substitute for it. Identification of impurities in new drug substances is likewise required above defined regulatory thresholds [28], and in practice a proposed structure is usually confirmed by synthesis or isolation. The checklist presented here is intended to make the gap between a computational result and the application-specific requirement explicit, not to lower that requirement.

## 2. Advances in AI-Assisted Structure Prediction

### 2.1. Retrieval, Generation, and Hybrid Approaches

The first distinction is between a ranked answer within a supplied candidate set and a structure generated without requiring membership in that set. In this review, ‘AI-assisted’ refers to methods in which at least one step uses a model learned from data, such as learned fingerprints, spectral embeddings, neural spectrum simulators, and generative models; rule-based and combinatorial methods, such as fragmentation trees and in silico bond disconnection, are referred to as conventional computational methods. Both are discussed because they are routinely combined. Database-restricted retrieval evaluates candidate molecules drawn from a chemical structure database, frequently filtered by measured precursor mass or molecular formula. A model may use calculated fragment pathways, learned fingerprints, or similarity between measured and simulated spectra. Its top-ranked answer is conditional on candidate coverage: when the true structure is absent, even an apparently decisive ranking may favor the nearest available analogue [14,15,17,18].

Database-free generation constructs molecular representations, such as SMILES strings or molecular graphs, without forcing the completed structure to be selected from a predefined list. It can therefore propose previously unlisted constitutional structures, but ‘database-free’ does not mean free of chemical priors. The network was trained on available structures and spectral data; molecular formula, instrument metadata, or a substructure constraint may still be provided at inference. Absence of a generated structure from the searched database also does not show that it was absent from the training data or that it is a new compound; the training set and larger compound databases should be checked before novelty is claimed. Hybrid approaches first obtain a related scaffold or template and then generate a conditioned structure. These categories describe the final candidate space and provenance of structural information, not mutually exclusive model architectures [4,6,7,12,29,30].

The distinction has immediate consequences for reporting. A correct database rank, a correct molecular formula, a chemically valid generated graph, and an experimentally verified structure are separate achievements. A spectral embedding, predicted fingerprint, or simulated spectrum may support any of these routes, but is not itself a verified identification (Table 1). Model outputs should therefore be described using task-specific terms rather than a generic ‘AI identification accuracy’ [19,20,31]. The approaches also differ in maturity. Retrieval has been used and benchmarked for more than a decade, including in community contests [32], and remains the default when a relevant structure database exists; generative and hybrid approaches are recent, have mostly been evaluated on curated benchmarks, and are intended mainly for compounds absent from structure databases, which are often also poorly represented in their training data [19,31]. Table 1 also lists the inputs and priors typically supplied to each class of method.

### 2.2. Computational Methods for Structure Retrieval

The tools discussed below were selected as widely used and openly documented examples of each computational role, not as an exhaustive inventory, which is available elsewhere [18,20]. In a typical structure-retrieval workflow, precursor-ion and fragment-formula assignments constrain the candidate space before individual structures are scored. SIRIUS integrates accurate masses, isotope patterns, and fragmentation trees to evaluate molecular formulas; ZODIAC uses relationships across a dataset, while MIST-CF applies learned formula inference [16,33,34]. These methods provide essential input to database searching, although the same formula constraints can also guide de novo generation. An incorrect precursor formula can therefore misdirect both approaches. Errors at this stage propagate: an adduct assigned wrongly (for example, [M+Na]^+^ read as [M+H]^+^) or an in-source fragment taken as the precursor changes the neutral mass, so that the formula, the candidate list, and every downstream score refer to the wrong molecule, while the model may still return a confident-looking answer. The reliability of the upstream assignment should remain visible in the final annotation.

Candidate ranking can then use observed reference spectra or computational explanations of fragmentation. MassBank and Global Natural Products Social Molecular Networking (GNPS) resources provide experimental spectra and related molecular-network information, but exact matching is constrained by library coverage [21,22,35,36,37,38,39]. Hybrid similarity search extends library matching to near-neighbor analogues by combining direct fragment matches with matches shifted by the precursor mass difference, an approach developed for illicit-drug identification [40]. MetFrag and MS-FINDER extend the search to molecular structures for which measured reference spectra may not be available, using in silico fragmentation and chemically informed rules to prioritize formula-compatible candidates [13,14,15]. These methods expand practical search coverage but cannot recover an analyte that has been excluded from the candidate list.

Machine learning adds structural descriptors to this retrieval process. CSI:FingerID estimates molecular fingerprints from MS/MS data and searches for compatible database structures; MIST predicts molecular fingerprints from formula-annotated fragment (subformula) representations [5,16,17]. CANOPUS predicts chemical classes rather than unique molecular connectivity, providing a useful alternative when isomeric structures cannot be discriminated [41]. COSMIC addresses confidence in computational annotations and reinforces the need to distinguish a high-ranked candidate from a confirmed compound identity [42]. The appropriate reporting level depends on both the model output and the structural alternatives remaining in the search space.

Additional computational tools support candidate comparison rather than defining a separate route to identification. Spec2Vec, MS2DeepScore, and DreaMS learn spectral relationships that can assist similarity searches and downstream inference [9,11,43]; spectral entropy and cross-ionization-mode methods offer other ways to compare spectra [44,45]. In the complementary forward direction, CFM-ID and neural spectrum predictors simulate expected fragmentation from a proposed structure, allowing predicted and measured ions to be compared [8,10,46]. These components can also inform generative pipelines, so they are not exclusive to retrieval. Crucially, agreement between two computational predictions does not constitute independent experimental confirmation when they share training data or chemical assumptions.

### 2.3. De Novo and Hybrid Structure Generation

Database-free generation addresses the limitations imposed by a fixed list of candidate structures. MassGenie, MSNovelist, and Spec2Mol illustrate approaches that translate spectral information into molecular representations, sometimes through predicted fingerprints or learned molecular embeddings [4,6,7]. DiffMS uses a formula-constrained graph diffusion strategy to propose candidate molecules [30]. MS-GPT converts the spectrum into multiple calibrated fingerprint hypotheses, uses them to query a pretrained molecule-language model conditioned on the molecular formula, and ranks the pooled candidates by generation frequency [47]. In contrast, hybrid approaches combine candidate retrieval with generation: MADGEN first retrieves a plausible scaffold and then generates structures conditioned on that scaffold and the spectrum, while MetGenX uses related structural templates to guide candidate generation and ranking [12,29]. The information supplied to a hybrid model can dominate its performance: on MassSpecGym, MADGEN reached 10.5% top-1 accuracy when given the correct scaffold, an oracle condition that marks an upper bound rather than routine practice, but 1.31% with its own predicted scaffold; on NIST23 the corresponding values were 49.0% and 4.6% [29]. Domain-specific impurity elucidation illustrates how chemically justified information can constrain such searches: the method of Dorigatti et al. requires the molecular formula of the impurity, accepts a known substructure of the drug substance as a prompt, and is fine-tuned on impurities predicted from the synthetic route with reaction templates [48]. As with a supplied scaffold, its accuracy is conditional on the correctness of these inputs.

The distinction between database-free and hybrid approaches concerns how the search is constrained, not whether a model has ever encountered chemical structures during training. Providing the correct molecular formula, a retrieved scaffold, or a known synthetic intermediate can substantially improve tractability, but this information must be reported as model input rather than credited to independent structural deduction from MS/MS. The typical failure modes follow from these constraints. When the supplied formula or scaffold is wrong, the generator searches the wrong region of chemical space and may still return chemically valid, spectrum-compatible molecules; when the spectrum is noisy, chimeric, or poor in informative fragments, the output is dominated by the learned prior, which favors common substructures; and generated molecules can be invalid, implausible for the sample, or close analogues of training structures [19,30,31]. Both generation routes therefore produce hypotheses requiring analytical discrimination, particularly when several isomers share the same informative fragments.

### 2.4. Performance Evaluation and Benchmarking

Retrieval, generation, and hybrid methods cannot be evaluated as if they were solved by an identical identification task. Database-restricted retrieval ranks members of a predefined candidate set, which may already include the correct answer. Database-free generation must instead construct molecular candidates without selecting from that set, while hybrid generation can exploit a supplied scaffold or related template. For retrieval, the CASMI 2016 contest compared annotation tools on blinded spectra; the best methods ranked the correct structure first for more than 30% of the 208 challenges using in silico fragmentation alone, and for about 70% when additional information, such as metadata, was allowed. Because candidate lists were supplied, these results assume that the correct structure is present [32]. MassSpecGym distinguishes molecular retrieval, de novo generation, and spectrum simulation in its evaluation framework and uses challenging data splits to assess generalizability [19,31]. Under this leakage-controlled split, DiffMS recovered the exact structure for 2.3% of MassSpecGym spectra at top-1 and 4.25% at top-10 [30], and higher accuracies reported on earlier GNPS splits were likely inflated by structural leakage [19]. A recent preprint reports 23.9% top-1 accuracy for MS-GPT under the same known-formula protocol [47]; such gains still require peer review and independent replication. Reported top-1 or top-k accuracy therefore needs to be interpreted in relation to the task, available candidate space, and structural information provided to the model.

A meaningful comparison should specify the training–test split, availability of reference spectra, candidate-set completeness, supplied formula or scaffold, and success criterion. Exact structural matches, correct formulas, class-level predictions, and molecular similarity are different endpoints. In this review, an exact structural match means identical molecular connectivity, corresponding to the first (connectivity) block of the InChIKey; a stereochemistry-aware match additionally requires identical stereodescriptors, that is, the full InChIKey. Benchmarks should state which definition is used; otherwise, generators that output stereo-free graphs are scored as wrong whenever the reference structure carries stereodescriptors. Duplicate or closely related structures across training and test sets, termed leakage between training and test data, may inflate apparent performance on unknown compounds. Because the formula of an uncharacterized feature is itself uncertain, accuracy obtained with the correct formula is best read as an upper bound, and practical performance also depends on how reliably the formula is assigned [12,16,19,20,29,30,31,33,34]. Benchmarking can establish comparative computational performance under defined conditions, but it cannot by itself establish the identity of an individual analyte. Table 1 therefore separates primary prediction strategies from computational components used across them, together with the inputs they typically require and the limits of the structural claim each output can support.

## 3. Sources of Uncertainty in AI-Assisted Structure Prediction

### 3.1. Analytical Limitations of MS/MS Evidence

The principal limitation is sometimes in the experiment rather than the prediction algorithm. Collision-induced dissociation favors only a subset of accessible pathways; shared functional groups or rearrangements may produce the same abundant fragments in different constitutional isomers. In-source fragmentation, adduct misassignment, and chimeric spectra arising from co-isolation or data-independent acquisition may add peaks not belonging to the intended molecule. A model cannot resolve connectivity that the measured ions do not differentiate. High mass accuracy improves confidence in elemental compositions but does not uniquely establish positional (regio-) or stereochemical identity [2,3,13,15,18].

Acquisition and processing settings determine what evidence reaches the model. A wide precursor isolation window or co-eluting isobaric ions produce chimeric spectra, which are frequent in data-dependent acquisition of complex matrices; precursor-purity assessment can flag spectra that are likely to be chimeric, and database-assisted deconvolution can separate the contributions of known compounds [49,50]. In-source fragments and adducts co-elute with their parent ion and can be mistaken for independent precursors or genuine metabolites, so that chromatographic separation from the possible parent is an essential check [51]; correlation of chromatographic peak shapes and variations in source conditions also help to recognize them. Collision energy and activation type change the relative abundance, and sometimes the presence, of diagnostic ions, and spectra acquired on different instrument platforms differ systematically; models trained mainly on one acquisition regime may perform less well on another [8,19,31]. Preprocessing choices, including intensity thresholds, centroiding, removal of the precursor peak, and intensity weighting, also change the input to which similarity scores and learned models respond [45]. These settings should be reported with the annotation because they bound its reliability.

Low-intensity diagnostic ions and different collision energies may be decisive even when their influence on a global similarity score is small. Therefore, an overall spectral score should be accompanied by inspection of unexplained major ions, fragment formulas, and ions that would be expected for competing hypotheses. Contradictory peaks can be more informative than additional peaks shared by every candidate.

### 3.2. Candidate-Space Bias and Overconfidence

In closed candidate retrieval, the winning candidate can be wrong because the true analyte is missing; in generation, an incorrect structure can be chemically plausible because the learned molecular prior favors common substructures. Both methods may agree on a familiar scaffold while disagreeing on a substitution position. Furthermore, four different quantities are often conflated. A similarity score, such as a cosine or entropy similarity, measures agreement between two spectra; a ranking confidence, such as the score margin between the first and second candidates, describes order within one candidate list; a calibrated probability is a score shown on independent data to match the observed frequency of correct answers; and an identification confidence level, such as Schymanski Level 1–5, states which structural claim the total evidence supports [2]. Only the last is a reporting category for chemical identity. A cosine similarity of 0.90 does not mean 90% certainty. Error rates for a set of annotations can be estimated with decoy spectra or decoy structures, which yield false discovery rates [52,53], or with purpose-built confidence scores such as COSMIC [42]; a probability for an individual candidate additionally requires post hoc calibration of model outputs on held-out data [54]. Each such estimate is valid only for a population and error definition resembling the new analytical application [23,24,25].

False certainty is particularly consequential for unexpected synthetic drug analogues, unknown metabolites, or environmental transformation products because precisely these compounds may be poorly represented in training and candidate databases. The analyst should distinguish uncertainty in the rank order from uncertainty in the structural features actually supported by the data. In practice, this means examining what the leading candidates have in common: if the five highest-ranked candidates share a scaffold but differ in the position of a substituent, then the scaffold is supported and the position is not, and the report should say so. Class predictions such as CANOPUS can make this explicit [41]. A class-level claim may remain sound even when the top positional isomer is not.

### 3.3. Nonindependence of Computational Evidence

Apparent confirmation may be circular. Two computational tools may produce matching proposals without supplying independent evidence when they share training libraries or structure databases, use the same molecular descriptors or fingerprints, encode the same fragmentation rules or assumptions, or build on the same pretrained spectral or molecular representations [9,11,31,43]. Agreement between a neural spectrum simulator and a neural structure predictor may likewise reflect shared assumptions. Whether two tools are independent can usually be judged only from their documentation of training data and representations, which should therefore be consulted and reported. Predicted retention time and collision cross section (CCS) can provide useful complementary constraints, but only measured properties constitute additional experimental observations; a second predicted value is not a second measurement [18,55,56,57,58].

For small molecules, how often such tools agree on the same incorrect structure has not, to our knowledge, been estimated systematically, but shared failures have been documented. In the CASMI 2012 contest, a precursor heuristic shared by MetFrag and MetFusion, which builds on MetFrag, assigned the wrong neutral mass in two challenges, in one of which the main ion was a water-loss fragment rather than [M+H]^+^, so that both tools searched for candidates of the wrong mass [59]. In CASMI 2016, no participating method ranked abietic acid first despite an informative spectrum, and the organizers noted that the closely similar isopimaric acid may explain this in part [32]. In a post hoc comparison on the CASMI 2016 data, four in silico fragmentation tools all ranked the correct structure outside the top 20 for the same 15 compounds, which the authors linked to spectra with few fragment ions; the best voting model (MetFragCL with CFM-ID) ranked the correct structure first for 22% of the training spectra, compared with 10–17% for the individual tools, and a voting model did not improve on MetFragCL alone for the challenge spectra [60]. Shared chemical assumptions can also make several resources wrong at once: in a small test of three dozen product ions from five precursors, nearly all fragment-ion structures annotated in three major in silico MS/MS libraries (HMDB, METLIN, and mzCloud) disagreed with the structures established by infrared ion spectroscopy, often because the fragmenters behind HMDB and METLIN, CFM-ID and MetFrag, neglect the cyclization rearrangements that formed many of the product ions [61]. Multi-tool strategies have become common, and agreement between tools is often treated as supporting evidence. When four tools were applied to experimental LC–HRMS features, full agreement was reached for only a small subset of compounds, top-ranked candidates were often chemically implausible or artifactual, and structures suggested by more than one tool were ranked above single-tool annotations, although reference standards were unavailable for most structures [62].

Shared training data and shared software components can make such failures correlated. Many learned tools are trained on the same public spectral libraries, and for a learned spectral similarity model trained on GNPS and MassBank data, retrieval ranking deteriorated as test compounds became less similar to the training structures [63]; tools trained on overlapping data may therefore fail on the same unfamiliar compounds. A recent preprint audit found evaluation problems in at least 17 of 26 studies reporting results on the MassSpecGym benchmark, including an erroneous checkpoint of a widely reused spectrum encoder that was carried into several de novo generation pipelines and training spectra simulated by a model that had seen the test data [64]. A direct estimate of shared errors is available for de novo peptide sequencing, a related MS/MS task: across eight deep learning models, incorrect predictions overlapped little between models, but most errors were model-independent and driven by limited fragment-ion coverage; all eight models returned the same sequence that disagreed with the database-search reference for only 0.174% of the spectra in one dataset, and 73% of these sequences were present in the search database, suggesting a chimeric spectrum or an incorrect reference [65]. The limited evidence suggests that complete agreement on a wrong answer is uncommon, but failures that arise from the shared spectrum or its interpretation, from shared training data, or from shared software components affect all tools alike and are not resolved by adding further computational tools.

A practical distinction is between evidence that increases the plausibility of a candidate and evidence that rules out a specific alternative. Accurate mass often sharply limits molecular formulas but does little to separate regioisomers. A measured retention-order difference, isomer-specific product ion, or authentic-standard comparison can be more decisive for a particular pair of candidates even when its contribution to a generic AI score is smaller. In weighing evidence, a single reproducible observation that contradicts a candidate, such as an expected diagnostic ion that is absent or a measured retention time outside the tolerance of an authentic standard, generally carries more weight than several observations that are compatible with all candidates. This makes the unresolved structural question, rather than a desire for one more score, the appropriate guide to validation.

## 4. Analytical Validation as Candidate Discrimination

### 4.1. Selection of Structure-Discriminating Experiments

We suggest a candidate-discrimination workflow organized around one question: which observable would change if the leading structure were false? First, verify the analytical feature and precursor assignment, including retention behavior, blank contamination, isotopes, and likely adduct or in-source fragment relationships; feature-processing environments such as MZmine 3 can facilitate transparent handling of multimodal data but do not by themselves establish chemical identities [66]. Second, retain several plausible structures from spectral libraries, database retrieval, and, when warranted, generation. Third, annotate which experimental features each candidate explains and which it fails to explain. The next measurement should be chosen to challenge the strongest competing explanations, not merely to reconfirm shared fragments [1,2,3,14,16,18]. The strongest competing explanations are those that explain the measured ions about as well as the leading candidate: candidates within a small score difference, positional isomers or stereoisomers of the leading structure, and structures from other sources with the same formula. Where only one tool is available, alternatives can be obtained by examining several top-ranked candidates rather than the first, by enumerating plausible positional isomers of the leading structure, and by consulting a class-level prediction. The following example illustrates the workflow for two competing isomers.

Consider a drug metabolite whose protonated molecule is 15.995 Da heavier than that of the parent drug, consistent with addition of one oxygen atom. Retrieval and generation both return two leading candidates that differ only in the site of hydroxylation, the aromatic ring (a phenol) or the adjacent alkyl chain (an alcohol); both explain the shared core fragments, and their scores are close. If the parent drug contains a tertiary amine or a pyridine-type nitrogen, then an N-oxide should be retained as a third candidate; like a phenol, it shows little water loss in electrospray MS/MS, but under atmospheric pressure chemical ionization it gives an [M+H−O]^+^ ion that hydroxylated isomers do not [67].

The observable that would change if the leading candidate were false is the loss of water and the location of the mass shift. An aliphatic hydroxyl group often gives a prominent loss of water from the precursor and from fragments that contain it, whereas a phenolic hydroxyl usually does not, as shown for loratadine metabolites [67]; in addition, the +15.995 Da shift should be carried by the product ions that contain the modified ring or the modified chain, respectively. The discriminating evidence is therefore multienergy MS/MS, which follows the water loss and the location of the mass shift, and, if a reference compound of either candidate is available, retention time under the same conditions.

The strongest supported claim then depends on the outcome. If the fragment evidence places the hydroxyl group on one specific carbon of the chain and no other structure fits the data, then a probable structure (Level 2b) can be reported. If the evidence locates the hydroxyl group only on the chain or only on the ring, for example, without resolving the ortho, meta, or para position, which commonly requires chromatographic comparison with standards [68], then the isomeric family (Level 3) is reported and the remaining positions are listed. A co-eluting authentic standard with a matching spectrum supports Level 1 [2]. Predicted retention times or CCS values can help to decide which standard to obtain first but do not by themselves decide between the candidates.

### 4.2. Evaluation of Evidence for Structural Assignment

Different measurements resolve different ambiguities. Precursor accurate mass and isotope patterns constrain composition; product-ion formulas and fragmentation networks support selected substructures; collision-energy series and alternative activation probe fragmentation mechanisms. Chromatographic retention is sensitive to polarity, hydrogen bonding, and stationary phase, while ion-mobility CCS provides a gas-phase shape-related measurement. Predicted retention times and CCS values can prioritize candidates and exclude implausible ones, but prediction uncertainty and overlapping values must be considered, and predicted values are not equivalent to experimentally measured orthogonal evidence; a candidate should not be assigned a higher confidence level on predicted values alone [55,56,57,58]. None of these properties automatically proves an exact molecular structure. When measurements conflict, for example when measured retention and CCS fit a candidate but a strong product ion is not explained, the conflict should be resolved by a further measurement or reported, not averaged away in a combined score.

When feasible, an authentic standard measured with a comparable method provides stronger evidence through agreement of retention and fragmentation behavior. It is not enough to assume that a commercial reference spectrum acquired under different conditions is equivalent to a same-method standard confirmation. Standards may be costly, unavailable, or obtainable only by custom synthesis, particularly for new metabolites and synthetic analogues; the remaining uncertainty should then be reported rather than resolved by a higher score, and shared spectra and candidate lists allow the identity to be confirmed later. For an unlisted compound lacking a standard, the report should explicitly say whether the evidence establishes molecular formula, chemical class, plausible connectivity, or a more restricted candidate family. In the framework of Schymanski et al. [2], Level 1 denotes a structure confirmed with a reference standard; Level 2 a probable structure supported by an unambiguous library or literature spectrum match (2a) or by diagnostic evidence when no standard or library spectrum is available (2b); Level 3 tentative candidates, for which the evidence supports a structure class or several possible structures; Level 4 an unequivocal molecular formula; and Level 5 an exact mass of interest. Level 2b requires that the diagnostic evidence leave no other structure that fits the data; a high AI score alone does not meet this condition. Table 2 indicates how each permissible claim corresponds to these levels, so that the checklist documents a level rather than redefining it. Established confidence frameworks remain useful; the proposed workflow is intended to help document why a particular level is justified, not to replace or relabel those schemes [1,2,23,24,25].

### 4.3. Documentation of Supporting and Contradictory Evidence

A concise candidate record can prevent analytical decisions from being obscured by software output. For each high-priority feature, record the raw MS/MS identifier, acquisition settings, precursor and formula alternatives, database and version, computational method and its inputs, the size of the candidate space, the top candidates with their scores (at least several, or all within a stated score margin, rather than only the best hit), diagnostic observed fragments, unexpected high-intensity ions, and which constraints were measured rather than predicted. Also, document which experimental feature could discriminate the top two candidates and whether it was obtained. A candidate with the best score but a strong contradictory fragment should not be presented as unqualified identification [25,38,39,52,53]. Table 3 gives a minimal template that can be placed as a table or spreadsheet with one row per annotated feature. The MSI reporting standards and the Schymanski levels define what each confidence level means [1,2]; the template adds what those schemes do not require, namely the computational inputs and priors and the evidence that discriminated, or failed to discriminate, the leading candidates.

Table 2 summarizes a minimal evidence-to-claim checklist. Its purpose is not to create another numerical confidence scale. Rather, it separates three different questions: (i) what did the algorithm calculate, (ii) what was actually measured, and (iii) which aspect of molecular structure does the combined information support? This distinction is portable across pharmaceutical impurity profiling, natural-product metabolomics, environmental non-targeted analysis, and forensic toxicology, even though the acceptable residual uncertainty may differ by application.

## 5. Conclusions and Perspectives

AI-assisted MS/MS structure prediction is advancing through database retrieval, learned representations, and increasingly capable generative and hybrid models. These developments expand the candidate structures available for interpretation and can guide efficient follow-up experiments. Nevertheless, retrieval accuracy depends on candidate coverage; generation accuracy depends on learned priors and supplied constraints; and neither automatically resolves isomeric ambiguity nor establishes a chemical identity [12,18,19,20,29,30].

For practical analytical chemistry, the next step is to make model outputs falsifiable, that is, to state for each proposed structure which observation would show it to be wrong and to acquire that observation where possible. Evaluations should report not only exact-match rates but also candidate-space assumptions, input priors such as a supplied formula or scaffold, the exact-match criterion including stereochemistry, calibration, and performance on genuinely novel analogues. A useful AI system may deliver a short, chemically coherent candidate set together with suggestions for the next experiment; this outcome can be scientifically valuable even when no exact name can yet be assigned. To our knowledge, current tools provide ranked candidates and class predictions but do not yet routinely propose discriminating experiments, which is a direction for development. Journals and spectral databases could support the distinction between prediction and identification by asking that annotations state the confidence level together with the tools, inputs, and evidence used (Table 3), and by labeling computationally derived annotations separately from experimentally confirmed ones.

In practice, we recommend that analysts (i) report model inputs and priors, including any supplied formula, scaffold, or database; (ii) retain and report plausible alternatives with their scores and the size of the candidate space; (iii) acquire discriminating evidence when possible, preferring measurements that could refute the leading candidate; and (iv) report only the strongest claim that the data support. The distinction between computational prediction and analytically justified identification should remain explicit in every application.

## Figures and Tables

**Table 1:** Computational approaches for MS/MS structure prediction and the analytical scope of their outputs.

Computational Role	Representative Methods and Resources	Primary Output	Main Limitation	Typical Inputs and Priors
Formula and class inference (shared)	SIRIUS; ZODIAC; MIST-CF; CANOPUS	Molecular formula or chemical-class hypotheses	Does not uniquely establish molecular connectivity	Precursor *m/z*, isotope pattern, MS/MS; element and adduct assumptions
Database-restricted retrieval	Libraries: MassBank, GNPS. Methods: NIST hybrid search; MetFrag; MS-FINDER; CSI:FingerID; MIST	Measured spectral match or ranked candidate structures	Requires relevant reference data or inclusion of the correct database candidate	MS/MS; precursor mass or formula; spectral library or structure database
Spectral similarity (shared)	Spec2Vec; MS2DeepScore; DreaMS; spectral entropy	Similarity measures, embeddings, related spectra	Similarity is not a calibrated probability of exact identity	Spectrum pairs; trained embedding or similarity function
Forward prediction (shared)	CFM-ID; graph-based spectrum predictors	Simulated MS/MS spectra for proposed structures	Not an independent experimental measurement	Candidate structure; collision energy and instrument type
Database-free generation	MassGenie; MSNovelist; Spec2Mol; DiffMS; MS-GPT	Generated candidate structures, often with constraints	Chemical validity or plausible fragments do not confirm exact identity	MS/MS; often molecular formula; learned chemical prior
Hybrid retrieval–generation	MADGEN; MetGenX; impurity-specific approaches	Candidates conditioned on scaffolds, templates, or domain priors	Results depend on the reliability of supplied structural constraints	MS/MS; often molecular formula; scaffold, template, or synthesis knowledge

All outputs in this table are computational, except a match to an experimental reference spectrum in a spectral library, which supports at most a probable structure (Level 2a). None of these outputs alone establishes chemical identity; confirmation requires an authentic standard analyzed under comparable conditions (Level 1) [2].

**Table 2:** A concise evidence-to-claim checklist for AI-assisted structure annotation.

Unresolved Analytical Question	Most Informative Check	Strongest Claim If Ambiguity Remains (Level [2])
Is the precursor formula/ion correct?	Isotopic pattern, adduct series, fragment-formula consistency	Feature- or formula-level annotation (Level 4–5)
Is the top-ranked hit missing the true structure?	Broaden candidate sources; retain generative alternatives	Computational ranking; tentative candidate set (Level 3)
Do top candidates share all major fragments?	Examine diagnostic ions, multienergy MS/MS, MS^n^	Structural-class or isomeric-family assignment (Level 3)
Can orthogonal separation distinguish between candidates?	Measured retention time or order and/or CCS with uncertainty	Tentative identification; prioritized candidate (Level 3); Level 2b only if measured diagnostic evidence excludes all other candidates
Are confidence scores meaningful for this sample?	Check split, calibration, domain, decoys, database and priors	Categorical confidence; no unsupported percentage (any level)
Is a unique identity required?	Authentic standard under comparable conditions; further structure-specific tests	Standard-confirmed identity (Level 1); otherwise state remaining uncertainty

Levels after Schymanski et al. [2]: 1, confirmed structure (reference standard); 2a, probable structure by unambiguous library or literature spectrum match; 2b, probable structure by diagnostic evidence when no other structure fits the data; 3, tentative candidate(s); 4, unequivocal molecular formula; 5, exact mass of interest. Predicted retention time or CCS values alone do not raise the level.

**Table 3:** Minimal reporting template for an AI-assisted structure annotation (one row per feature).

Item	What to Record	Example Entry
Feature	Precursor *m/z*, retention time, adduct, raw file and scan identifier	*m/z* 304.1543, 6.42 min, [M+H]^+^, file 07, scan 2381
Acquisition	Instrument type, polarity, isolation width, collision energies	Orbitrap, positive, 1.0 *m/z*, stepped 20/40/60
Formula	Assigned formula and alternatives with scores	C17H21NO4 (top); one alternative within 5 ppm
Tools and inputs	Software and model versions; database and version; supplied formula, scaffold, or other priors	Tool, version; database snapshot; formula supplied
Candidate space	Number of candidates searched or generated	1240 formula-compatible candidates
Top candidates	Several leading candidates (or all within a stated margin) with scores and ranks	Five candidates; score margin of first over second
Diagnostic evidence	Ions or measurements that discriminate the leading candidates; contradictory ions	Water loss present; unexplained ion at *m/z* 150.0913
Measured orthogonal data	Measured retention, CCS, or standard comparison, kept separate from predicted values	Standard not available; predicted retention time used for prioritization only
Reported claim	Confidence level and remaining alternatives	Level 3; two positional isomers remain

## Data Availability

No new data were created or analyzed in this study. Data sharing is not applicable to this article.
